# Mutations in CCNB3 affect its location thus causing a multiplicity of phenotypes in human oocytes maturation by aberrant CDK1 activity and APC/C activity at different stages

**DOI:** 10.1186/s13048-023-01229-8

**Published:** 2023-08-28

**Authors:** Congjing Wang, Meng Xi Chen, Yuan Zhang, Xue Bai, Qiqi Cao, Jian Han, Nana Zhang, Chun Zhao, Xiufeng Ling, Ximan Rui, Yichun Guan, Junqiang Zhang, Ran Huo

**Affiliations:** 1 State Key Laboratory of Reproductive Medicine and Offspring Health, Department of Histology and Embryology, Suzhou Municipal Hospital, Gusu School, Suzhou Affiliated Hospital of Nanjing Medical University, Nanjing Medical University, Nanjing, China; 2 Department of Reproductive Medicine, Nanjing Maternity and Child Health Care Hospital, Women’s Hospital of Nanjing Medical University, Nanjing, Jiangsu China; 3 Clinical Center of Reproductive Medicine, State Key Laboratory of Reproductive Medicine, First Affiliated Hospital, Nanjing Medical University, Nanjing, China; 4 Center for Reproductive Medicine, The Third Affiliated Hospital of Zhengzhou University, Zhengzhou, China; 5 Center for Global Health, School of Public Health, Nanjing Medical University, Nanjing, China

**Keywords:** Oogenesis, Female infertility, Cyclin B3, Anaphase-promoting complex/cyclosome

## Abstract

**Background:**

Oocyte maturation arrest results in female infertility and the genetic etiology of this phenotype remains largely unknown. Previous studies have proven that cyclins play a significant role in the cell cycle both in meiosis and mitosis. Cyclin B3 (CCNB3) is one of the members of the cyclin family and its function in human oocyte maturation is poorly understood.

**Methods:**

118 infertile patients were recruited and WES was performed for 68 independent females that experienced oocyte maturation arrest. Four mutations in *CCNB3* were found and effects of these mutations were validated by Sanger sequencing and in vitro functional analyses.

**Results:**

We found these mutations altered the location of cyclin B3 which affected the function of cyclin dependent kinase 1 (CDK1) and led to mouse oocyte arrested at germinal vesicle (GV) stage. And then, low CDK1 activity influenced the degradation of cadherin 1 (CDH1) and the accumulation of cell division cycle 20 (CDC20) which are two types of anaphase-promoting complex/cyclosome (APC/C) activators and act in different stages of the cell cycle. Finally, APC/C activity was downregulated due to insufficient CDC20 level and resulted in oocyte metaphase I (MI) arrest. Moreover, we also found that the addition of PP1 inhibitor Okadic acid and CDK1 inhibitor Roscovitine at corresponding stages during oocyte in vitro maturation (IVM) significantly improved the maturation rates in CCNB3 mutant cRNAs injected oocytes. The above experiments were performed in mouse oocytes.

**Conclusion:**

Here, we report five independent patients in which mutations in *CCNB3* may be the cause of oocyte maturation arrest. Our findings shed lights on the critical role of *CCNB3* in human oocyte maturation.

**Supplementary Information:**

The online version contains supplementary material available at 10.1186/s13048-023-01229-8.

## Background

The process of successful human reproduction commences with the fusion of a metaphase II oocyte and a sperm cell, resulting in the formation of a fertilized egg [1]. The application of in vitro fertilization (IVF) and intracytoplasmic sperm injection (ICSI) techniques has enabled many infertile couples to conceive and give birth to their own children [2]. Nevertheless, repeated IVF and/or ICSI failures may occur due to oocyte maturation arrest, which results in a significant number of women being unable to conceive. It has been estimated that approximately 8.6–15.2% of all infertility patients produce at least one meiotically incompetent oocyte [3]. Certain genes have been identified as potential contributors to oocyte maturation arrest, such as *TUBB8* mutations which can result in female infertility due to arrest at metaphase I, fertilization, or early embryonic development [1, 4]. Recent researches have also linked PATL2 and TRIP13 to human oocyte maturation [5, 6]. However, it is still largely unknown that the genetic etiology of most patients with oocyte maturation arrest [6].

**Fig. 1:**
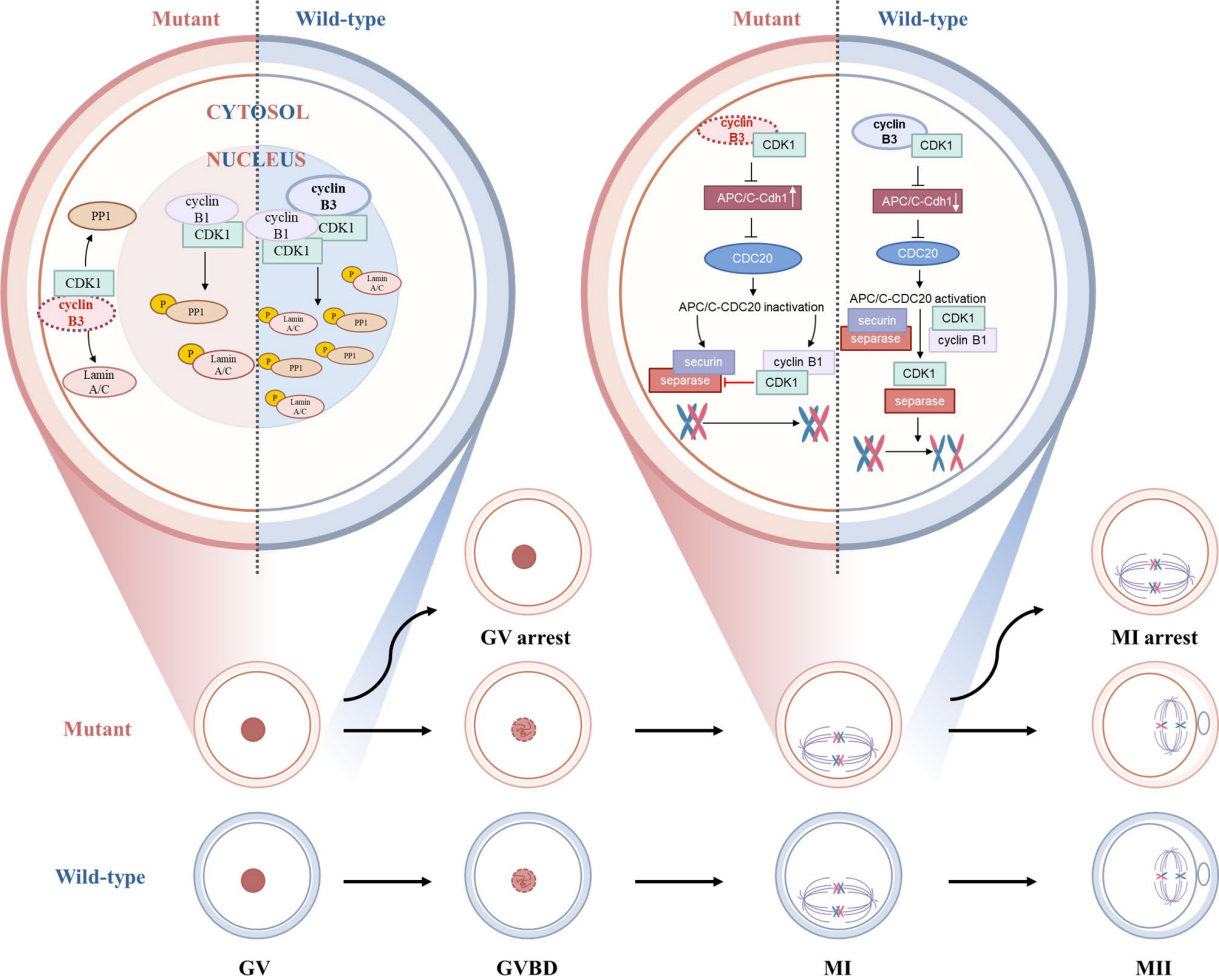
A schematic representation of the proposed mechanism by mutations in CCNB3 impairs oocyte meiotic process. The mutations affect the location of CCNB3 and affect the CDK1 activity in oocytes, resulting in partial oocytes arrest at the GV stage. Then, decreased CDK1 activity promote the level of APC/C-CDH1 and induce insufficient APC/C-CDC20 activity. Finally, the mutations cause oocyte meiotic arrest at metaphase I

Cyclins collaborate with cyclin-dependent kinases (CDKs) to facilitate the process of meiosis and mitosis through protein synthesis and degradation, thereby playing a crucial role in the cell cycle [7, 8]. A-type cyclins are integral to the progression of mitosis, while B-type cyclins are essential for regulating meiosis [9–11]. Previous studies have indicated that cyclin B1 and CDK1 are constituents of the Maturation Promoting Factor (MPF), which triggers meiotic resumption and regulates the progression of the cell through M-phase. With the resumption of meiosis, cyclin B1 gradually accumulates and it increases CDK1 activity by binding to CDK1. The activity of CDK1 reaches to maximum at 3 h after meiosis resumes. MPF activity is controled by APC/C at various stages. There are two types of APC/C activators, CDC20 and CDH1, that determine the substrate specificity of APC/C and play a role in corresponding period. Before the resumption of meiosis, oocytes maintain at the GV stage by degrading cyclin B1 and thus maintaining the activity of the kinase CDK1 at a low level. With the resumption of meiosis, the kinase activity of CDK1 is gradually rising with the accumulation of cyclin B1 and gradually switching off the APC/C-CDH1 activity by degrading CDH1. During prophase and prometaphase, the substrate specificity of APC/C-CDH1 is switched to degraded CDC20 and evevntuallly the degradation of CDH1 allows the activation of APC/C-CDC20. When APC/C-CDC20 becomes fully activated, cyclin B1 and securin are degraded and separase cleaves the cohesion ring, in parallel, the activity of CDK1 decreased due to lack of cyclin B1 (Fig. 1). Finally, the segregation of chromosomes initiates anaphase I [12]. However, in this study, mutant CCNB3 causes cyclin B1 to fail to degrade during MI. If cyclin B1 is not degraded in time, it keeps activating CDK1, which in turn leads to the maintenance of MI stage. The use of CDK1 inhibitor roscovitine could rescue PB extrusion and chromosome segregation.

Deletion of CDK1 from mouse oocytes results in the arrest at the GV stage [13]. Due to lacking the kinase activity of CDK1, the downstream protein phosphatae 1 (PP1) was not phosphorylated that led to an unphosphorylated state of Lamin A/C. PP1, a serine/threonine phosphatase, regulates a number of cellular processes by dephosphorylating its substrates and one of its substrates is Lamin A/C. The major function of unphosphorylated Lamin A/C is to maintain the structural integrity of the nuclear envelope [13]. Thus, the phosphorylated state of Lamin A/C plays an important role in oocyte maturation. When culture CDK1-/- oocytes with the PP1 inhibitor okadaic acid, germinal vesicle breakdown (GVBD) is largely resumed.

Undoubtedly, different cyclin/CDK1 complexes likely play different roles in the meiotic maturation of oocytes. However, with the exception of cyclin B1, the roles of specific cyclins in determining of ordered meiotic progression remain poorly understood in mammalian oocytes.

Cyclin B3 shares homology with A- and B-type cyclins and is conserved during the higher evolution of eukaryotic, implying that Ccnb3 may have acted in both meiotic and mitotic regulation. Previous studies have shown that Ccnb3 regulates meiosis cell cycle in Drosophila [14, 15]. In mice, cyclin B3 also plays a crucial role during oocyte maturation because knockout cyclin B3 in oocytes lead to MI arrest. Besides, the oocytes with overexpression of cyclin B3 cannot maintain at MII arrest [16, 17]. Nevertheless, oocytes with overexpression of cyclin B3 cannot maintain at MII arrest [18]. All of the findings indicate that *CCNB3* may be a candidate gene that contributed to oocyte maturation.

Here, we recruited 118 patients who experienced oocyte maturation arrest were recruited and then screened for oocyte maturation rate, 68 patients of them with oocyte maturation rate less than 40% were selected. To study its pathogenesis, these patients were sequenced by whole-exome sequencing (WES). Using a series of filters, we identified four heterozygous missense variants of *CCNB3* (GenBank NM_033031) that may be responsible for failed oocyte maturation in five affected individuals. We further studied the molecular mechanism of the corresponding pathogenic variants with HEK-293T cells and mouse models. Our findings showed that *CCNB3* mutations influenced the location of cyclin B3, followed by aberrant CDK1 activity that caused GV arrest. In turn, the low activity of CDK1 affected the degradation of CDH1. The high level of CDH1 kept degrading CDC20 and maintained a low level of CDC20, while the MI phase of normal oocytes require a high level of CDC20 to activate APC/C and promote the process of meiosis. In addition, in this study, we explored a potential value of the PP1 inhibitor, okadaic acid, and the CDK1 inhibitor, roscovitine, to rescue oocyte development arrest at the corresponding stages. Our findings could provide a potential marker of diagnostic significance for patients with primary infertility caused by oocyte maturation disorder and suggest a therapeutic intervention for the disease.

## Methods

### Human subjects and ethical approval

Individuals with primary infertility due to oocyte maturation arrest were recruited from the Reproductive Medicine Center of Women’s Hospital of Nanjing Medical University, First Affiliated Hospital of Nanjing Medical University, and The Third Affiliated Hospital of Zhengzhou University. All samples from donors were obtained with informed consent.

### Genomic DNA extraction, whole-exome sequencing, data analysis, and target gene sequencing

All genomic DNA samples from patients were extracted from peripheral blood with a RelaxGene Blood DNA System using standard methods (Tiangen, Beijing, China, DP319). Whole-exome sequencing was performed to identify candidate variants. Whole-exome capture used the Aglient SureSelect Human All Exon V6 (Agilent), and sequencing was carried out on the Illumina NovaSeq 6000 platform (Illumina) by Microanaly Genetech Co., Ltd. (Anhui).

118 patients were recruited and whole-exome sequencing was performed for 68 independent infertile females who suffered from experienced oocyte maturation arrest and their oocyte maturation rate below 40%. Candidate variants were filtered with the following criteria: (1) variants in coding regions; (2) variants with a minor alle frequency (MAF)>0.01 in gnomAD_EAS or ExAC_EAS were excluded; (3) patients with genes have reported are excluded; (4) variants in the genes that did not affect fertility according to the literature were excluded; (5) variants in gene had a population cluster are kept. Sanger sequencing was performed to verify mutations of CCNB3 in all patients. Primers used for CCNB3 exon sequencing were provided in Table 1.

**Table 1:** Primers

Primer name	Primer sequence
E116A-F	TAAGCTGGCAGTCACACCAGTAGTAGCCTCTACTACC
E116A-R	GTGTGACTGCCAGCTTATGCCATTTTAGATTCC
E221A-F	TAAAACTGAGGCGGCAGCCATCACCAAGAAGA
E221A-R	CTGCCGCCTCAGTTTTATGTGTCTTCTTAAAAGTC
P1223L-F	CAACTCACTTCGTGTGGATGACTTTGTGTACATC
P1223L-R	CCACACGAAGTGAGTTGTGCTCCTCAAATTTTG
S1353N-F	CGATAATCTCAAGGCTGTGTATTACAAGTATTCT
S1353N-R	CAGCCTTGAGATTATCGTAAGAACTGAAAGTCAGCAGTT

### Expression constructs and in vitro transcription

Wild-type human CCNB3 were constructed and then recombined with the eukaryotic expression vector pcDNA3.1. A FLAG-tag was fused at the C-terminus of CCNB3. The vectors were constructed by GenScript (Nanjing). The variants were generated using Fast Mutagenesis Kit (Vazyme, Nanjing, C214). The plasmids were linearized with ApaI enzyme (New England Biolabs, # R0114S) and then be transcribed to cRNAs using HiScribe T7 ARCA mRNA Kit (New England Biolabs, E2060) according to the manufacturer’s standard mRNA synthesis protocols.

### Cell culture and transfection

HEK-293T cells were maintained in Dulbecco’s modified Eagle’s medium (DMEM, Life Technologies/Gibco, Grand Island, New York, #11,995,073) supplemented with 10% fetal bovine serum (FBS) (Life Technologies/Gibco, Grand Island, NY, #10,270,106), 10,000 units/ml of penicillin and 10,000 µg/ml of streptomycin (Invitrogen, 15140-122), and the cells were cultured at 37 °C with 5% CO2. Cells were transiently transfected for 6 h using Lipofectamine 2000 reagent (Invitrogen, USA, #11,668,019), then cells were washed twice with PBS and maintained in serum-free medium for 48 h before harvesting.

### Western blotting

Cell protein concentrations were determined with a BCA Protein Assay (Beyotime Biotechnology, China, P0012). Oocyte protein concentrations were adjusted by the equal number of oocytes (A hundred oocytes were used for each lane). The protein was separated by 10% sodium dodecyl sulfate-polyacrylamide gel electrophoresis before being transferred to polyvinylidene fluoride membranes (Millipore, Massachusetts, IPVH00010). Non-specific binding sites were blocked for 2 h at room temperature with 5% non-fat milk in Tris-buffered saline containing 0.05% Tween-20. Membranes were incubated overnight at 4 °C with a dilution of the following antibodies: GAPDH (Abclonal, China, AC002 1:10000), FLAG (SIGMA, USA, F7425 1:1000), phospho-PP1α (Thr320)(Cell Signaling Technologies, USA, #2581,1:250), phosphor-Lamin A/C(Ser22) (Cell Signaling Technologies, USA, 13448,1:250), CDC20 (Santa Cruz, sc-13162,1:500), PTTG (Santa Cruz, sc-56,207 1:500), Cyclin B1 (Proteintech, 55004-1-AP, 1:1000), E-Cadherin Polyclonal Antibody (Proteintech, 20874-1-AP, 1:1000). After incubation with an anti-immunoglobin horseradish peroxidase-linked antibody (Invitrogen, USA, #31,430 and #31,460) for 1 h, the immune complexes were detected by enhanced chemiluminescence (FDBIO, China, FD8020). For densitometric analyses, protein bands on the blots were measured by ImageJ software.

### Immunofluorescence and confocal microscopy

Oocytes or HEK-293T cells were fixed in phosphate-buffered saline (PBS) supplemented with 4% paraformaldehyde (PFA) (Sigma, P6148) for 30 min at room temperature and then incubated in 0.5% Triton X-100 for 30 min at 37 °C. Then, samples were blocked in 1% bovine serum albumin in PBS for 1 h and incubated with primary antibodies overnight at 4 °C with a dilution of the following antibodies: FLAG (SIGMA, USA, F7425 1:500) and PTTG (Santa Cruz, sc-56,207 1:50) and CDK1 (Proteintech, 19532-1-AP, 1:50). After being washed three times in PBS, the samples were incubated with secondary antibodies for 1 h at room temperature. After washed three times in PBS, the samples were stained with Hoechst 33,342 (KeyGen BioTECH, KGA212-10) for 10 min and observed by confocal microscopy (LSM 800; Carl Zeiss, Germany). At least 40 oocytes were examined in each treatment in the experiment to detect the position of CDK1.

### Mouse oocyte collection and microinjection

Female ICR mice (4 weeks) were used for oocyte collection. To collect fully grown GV oocytes, mice were superovulated by intraperitoneal injection with 5 IU pregnant mare serum gonadotropin (PMSG) (Ningbo Sansheng Pharmaceutical Corporation, Zhejiang, China). Cumulus-enclosed oocytes were obtained by manual rupturing of antral ovarian follicles 48 h later. To obtain fully grown GV oocytes, cumulus cells were removed by repeatedly pipetting. For in vitro maturation, GV oocytes were cultured in M16 medium (Sigma, M7292) under mineral oil (Sigma, M8410) at 37 °C in a 5% CO2 incubator. For microinjection, fully grown GV oocytes were harvested in M2 medium (Sigma, M7167) with 2.5 µM milrinone (Sigma, M4659) to inhibit meiotic resumption. Approximately 10 pL of complementary RNA was injected at a concentration of 1,000 ng/µl. After injections, oocytes were arrested at the GV stage in M2 medium containing 2.5 µM milrinone for 12 h to allow sufficient translation, then washed in milrinone-free M16 medium, and cultured for 3 h to observe meiotic resumption (GVBD) or 14 h to detect PB1 extrusion.

For detecting the activity of CDK1 during the resumption of meiosis, *CCNB3* WT or mutant cRNA into GV oocytes. After injections, oocytes were arrested at the GV stage in M2 medium containing 2.5 µM milrinone for 12 h to allow sufficient translation, then washed in milrinone-free M16 medium, and cultured for 3 h for further study.

For detecting the activity of CDK1 and APC/C-CDC20 activity during the MI to MII transformation, *CCNB3* WT or mutant cRNA into GV oocytes. After injections, oocytes were arrested at the GV stage in M2 medium containing for 10 h, then washed in milrinone-free M16 medium, and cultured for 10 h for further study.

For inhibitor studies, the PP inhibitor OA (2 µM) was used in M16 medium and roscovitine (0.2 mM) was used in M16 medium. GV oocytes were injected with cRNA and then were arrested in M2 medium containing milrinone for 12 h, then washed in milrinone-free M16 medium, and cultured for 3 h in M16 medium containing okadaic acid. In another set of experiments, GV oocytes were injected with cRNA and then were arrested in M2 medium containing milrinone for 12 h, then washed in milrinone-free M16 medium, and cultured for 10 h in M16 medium. Finally, these oocytes culture for 3 h in M16 medium containing roscovitine.

### Controlled ovarian stimulation (COS) protocol

For patient 1, in the first cycle, the daily dose of recombinant FSH was 112.5 IU. After the first 4 days of treatment with recombinant FSH, the dose was increased to 125 IU for 2 days. Then, the dose was increased to 150 IU for 5 days. The patient was injected HCG with 2000 IU in the last day. The trigger time is 36 h. In the second cycle, the daily dose of Clomiphene was 50 IU, the daily dose of HMG was 10 IU for 9 days. On the tenth day, the patient was injected HCG with 4000 IU. The trigger time is 36 h. In the third cycle, during the first 8 days of treatment, the daily dose of recombinant FSH was 150 IU. On the ninth day, the dose of recombinant FSH was 50 IU and the dose of HMG was 150 IU. On the tenth day, the dose of HMG was 225 IU. On the eleventh day the patient was injected HCG with 2000 IU. The trigger time is 36 h.

For patient 2, during the first 4 days of treatment, the daily dose of recombinant FSH was 175 IU. After the first 4 days of treatment with recombinant FSH, the dose was increased to 200 IU for 2 days. Then, the next 6 days the daily dose of recombinant FSH was 225 IU and the daily dose of HMG was 75 IU. The patient was injected HCG with 5000 IU in the last day. The trigger time is 36 h.

For patient 3, during the first 4 days of treatment, the daily dose of recombinant FSH was 200 IU. After the first 4 days of treatment with recombinant FSH, the dose was increased to 225 IU for 2 days. Then, the next 6 days the daily dose of recombinant FSH was 225 IU and the daily dose of HMG was 75 IU. The patient was injected HCG with 4000 IU in the last day. The trigger time is 36 h.

For patient 4, during the first 5 days of treatment, the daily dose of recombinant FSH was 275 IU. Then, the next 2 days the daily dose of recombinant FSH was 200 IU and the daily dose of HMG was 75 IU. The patient was injected HCG with 5000 IU in the last day. The trigger time is 36 h.

For patient 5, the daily dose of recombinant FSH was 300 IU for 12 days. The patient was injected HCG with 5000 IU in the last day. The trigger time is 36 h.

### Statistical analyses

Statistical differences between groups were conducted by Student’s t-test and a one-way ANOVA when appropriate. Derived values are presented as the means ± SEM p < 0.05 were considered statistically significant. “n.s.” refers to non-significant.

## Results

### Clinical characteristics of the affected individuals

**Fig. 2:**
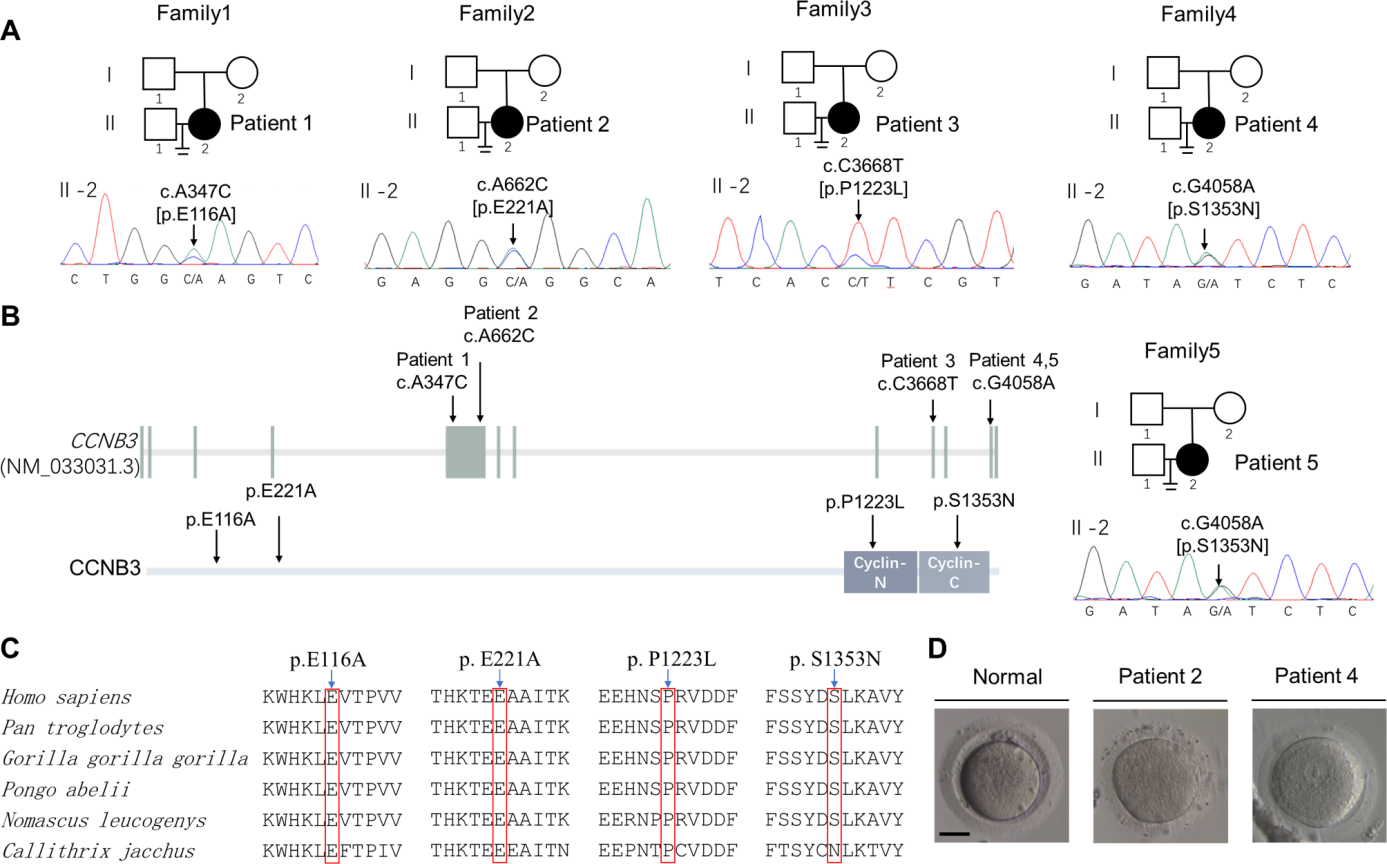
Identification of mutations in CCNB3. **(A)** Each affected individuals carries a heterozygous mutation; Sanger sequencing confirmation is shown below the pedigrees. Black circles indicate affected individuals. The “=” sign indicates infertility. **(B)** Location and conservation of mutations in CCNB3. The positions of E116A and E221A mutations are indicated in exon 5, P1223L located in exon 9 and S1353N located in exon 11. The patient 1 carried mutation c.A347C p.E116A, patient 2 carried mutation c.A662C p.E221A and patient 3 carried mutation c.C3668T p.P1223L. Patient 4 and patient 5 carried the same mutation c.G4058A p.S1353A. **(C)** The conservation of the mutations among multiple species. **(D)** Morphology of oocytes retrieved from control individuals and patients. The scale bar represents 40 μm

118 patients with oocyte maturation disorder were recruited from reproductive medicine centers. Among them, 68 patients with oocyte maturation rates below 40% (68/118) were selected for whole-exome sequencing, which was conducted using genomic DNA extracted from patient blood. Following a series of filtering procedures, as described in the Material and Methods, four *CCNB3* mutations were identified in five patients (Supplemental Fig. 1). All of the affected individuals had experienced primary infertility for several years, despite having normal menstrual cycles (Table 2). Each patient underwent at least one failed IVF and/or ICSI attempt. Affected patient 1 had undergone one failed IVF attempt and two failed ICSI attempts. Overall, 41 oocytes were retrieved, 6 oocytes were arrested at GV stages, 3 oocytes were arrested at MI stage, 9 oocytes were abnormal and cannot recognize the stage, 23 first polar bodies were retrieved, but 16 of them cannot be fertilized, and the remaining were fertilized. However, all fertilized oocytes were arrested at the 4–9 cell stage on day 3 (Table 3). In another independently recruited case (patient 2), 6 oocytes were retrieved in one IVF cycle, of which 1 oocyte was arrested at GV stage, 4 oocytes were arrested at MI stage and the remaining 1 oocyte extruded first polar body but cannot be fertilized (Fig. 2D; Table 3). A third patient was diagnosed with primary infertility of unknown cause and had a failed IVF attempt, 14 oocytes were retrieved and all of them were arrested at MI stage (Table 3). Patient 4 had undergone one IVF attempt and 5 oocytes were retrieved, including 4 MI arrested oocytes and 1 fertilized oocyte which cannot cleavage (Fig. 2D; Table 3). For patient 5, who had experienced one failed IVF attempt and one failed ICSI attempt, a total of 21 oocytes were retrieved, of which 15 were arrested at MI stage, the remaining fertilized but cannot cleavage (Table 3). Combined with a clinical classification scheme for recurrent oocyte maturation failure (ROMA) which defined by Beall,[3] we classified the phenotypes of these five patients. The patient 3 is type II whose oocytes all arrested before the first polar body (PB1) formation. While other patients are type IV for that they produced oocytes that arrested at more than one stage of meiosis.

**Table 2:** Medical history and laboratory investigation for patients

Patient	Age	Duration of Infertility(Years)	BMI	Basal sexual hormone			AMH (ng/ml)
				FSH (IU/L)	LH (IU/L)	E2 (pg/ml)	
Patient1	30	17	20	6.82	4.34	31	2.5
Patient2	34	3	24.8	5.66	1.52	128	9.76
Patient3	36	10	22.3	-	-	-	14.53
Patient4	28	2	18.4	13.59	3.18	31	3.05
Patient5	32	2	19.3	5.2	2.0	136.4	2.56

**Table 3:** Oocyte and embryo characteristics of IVF and ICSI attempts for the affected individual

Patient	Cycle								
	IVF/ICSI Cycles	Total Number of Oocytes Retrieved	GV Oocytes	MI Oocytes	MII Oocytes	Fertilized Oocytes	No. of unknown oocyte stage	Cleaved Embryos	OMF type
Patient1	1(IVF)	14	0	1	5	0	8	0	IV
	2(ICSI)	12	4	1	4	0	1	2	
	3(ICSI)	15	2	1	7	0	0	5	
Patient2	1(IVF)	6	1	4	1	0	0	0	IV
Patient3	1(IVF)	14	0	14	0	0	0	0	II
Patient4	1(IVF)	5	0	4	0	1	0	0	IV
Patient5	1(ICSI)	8	0	3	0	5	0	0	IV
	2(ICSI)	13	0	12	0	1	0	0	

IVF-in vitro fertilization; ICSI-intracytoplasmic sperm injection; PB1-first polar body; OMF type-oocyte maturation failure type.

**No. of unknown oocyte stage- some of the oocytes had multiple fragments and we cannot identify their specific periods**

### Identification of CCNB3 mutations

**Fig. 3:**
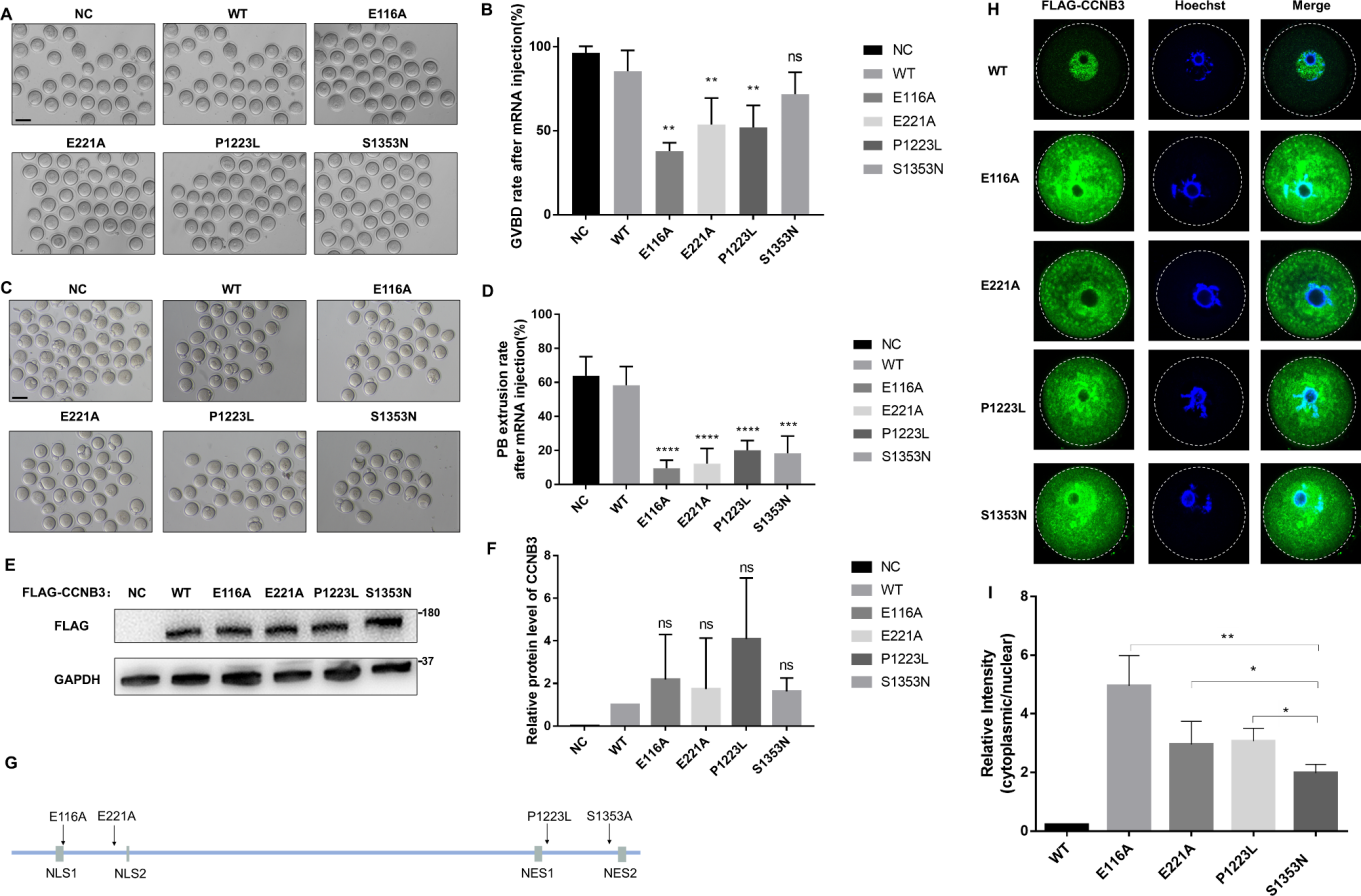
Mimicking the oocyte maturation arrest phenotype in mouse oocytes in vitro, effects of CCNB3 mutation on protein level and location. **(A)** Images of mouse GV oocytes injected with WT or mutant cRNAs and cultured for up to 3 h. **(B)** Quantitative analysis of GVBD rate. **(C)** Images of mouse GV oocytes injected with WT or mutant cRNAs and cultured for up to 13 h. **(D)** Quantitative analysis of PB1 rate. Graphs show means ± SEM of results observed in 3 independent experiments. **P < 0.01, **** p < 0.0001. **(E)** The effects of the mutations on CCNB3 protein level by Western blot in transfected HEK-293 cells. **(F)** Quantitative analysis of CCNB3 protein level. **(G)** Predict the domains of the CCNB3 protein, the positions of E116A and E221A mutations are closed to NLS and the positions of P1223L and S1353N are close to NES. NES: nuclear export signal; NLS: nuclear localization signal. **(H)** Immunofluorescence results of oocytes injected with CCNB3 (WT or mutant) cRNA; Blue: DAPI; Green: flag-WT or flag-mutated CCNB3. The scale bar represents 50 μm. Data are shown as the means ± SEM. For each kind of variant,n = 10 biological replicates. **(I)** Relative intensity of cytoplasmic/nuclear representing foci intensity

Based on the analysis of WES data and our filtering criteria (Supplemental Fig. 1, the details were also described in Material and Methods), we have identified four missense variants in *CCNB3* (GenBank: NM_033031), that are likely to be pathogenic. The positions of these mutations were shown in Fig. 2B. Patient 1 carried a missense mutation c.A347C (p.E116A). Patient 2 had a missense mutation c.A662C (p.E221A). The affected patient 3 had a missense variant c.C3668T (p.P1223L). The affected individuals of patient 4 and patient 5 had the same mutation site, c.G4058A (p.S1353N). All variants were confirmed by Sanger sequencing (Fig. 2A; Table 1). The inheritance pattern in the families was unknown due to the unavailability of DNA samples from their parents. Mutations in patient 1 c.A347C (p.E116A) and patient 2 c.A662C (p.E221A) have not been reported in any public databases, while variant c.C3668T (p.P1223L) in patient 3 has a low frequency of 0.0003 in ExAC_EAS and 0.0002 in gnomAD_EAS, variant c.G4058A (p.S1353N) in patient 4 and patient 5 has frequency of 0.0000777 in gnomAD_EAS (Table 4). The mutation assessments of the four variants in the SIFT, Polyphen2, CADD, REVEL and MutationTaster are shown in Table 4. Most of the affected residues are conserved across different species (Fig. 2C). Specifically, the variant c.A347C (p.E116A) was located in the cyclin-N box domain, while c.A662C (p.E221A) was located in the cyclin-C box domain (Fig. 2B). To further investigate the impact of these mutations on protein location, we utilized NetNES 1.1 Server and Moseslab to generate a predictor effect (Fig. 3G). Our findings indicated that CCNB3 contains two nuclear localization sequences (NLS) and three nuclear export signals (NES), and the four mutant amino acid residues of CCNB3 respectively corresponded to the nuclear localization signals (NLS, E116A and E221A) and the nuclear export signals (NES, P1223L and S1353N).

**Table 4:** CCNB3 pathogenic variants observed in 5 patients

Patient	cDNA change	Protein change	Mutation type	ExAC_EAS^a^	gnomAD_EAS^b^	Genotype	SIFT^c^	Polyphen2^c^	CADD ^c^	REVEL ^c^	MutationTaster ^c^
Patient1	c.A347C	p.E116A	Missense Mutation	NA	NA	Heterozygous	Damaging	Possibly damaging	18.27	0.109	Polymorphism
Patient2	c.A662C	p.E221A	Missense Mutation	NA	NA	Heterozygous	Damaging	Possibly damaging	19.06	0.171	Polymorphism
Patient3	c.C3668T	p.P1223L	Missense Mutation	0.0003	0.0002	Heterozygous	Damaging	Damaging	24.7	0.213	Disease causing
Patient4 and 5	c.G4058A	p.S1353N	Missense Mutation	NA	0.0000777	Heterozygous	Tolerated	Benign	0.003	0.051	Polymorphism

a Frequency of corresponding mutations in the East Asian population of the ExAC Browser.

b Frequency of corresponding mutations in gnomAD.

c Mutation assessment by Sift, Polyphen-2 (PPH2), CADD, REVEL, MutationTaster

NA-not available

### Mimicking the oocyte arrest phenotype in mouse oocytes in vitro

In order to establish a correlation between *CCNB3* mutations and the phenotype of oocyte maturation arrest, we generated both wild-type and mutant *CCNB3* (E116A, E221A, P1223L and S1353N) constructs, which were subsequently recombined with the eukaryotic expression vector pcDNA3.1 containing a FLAG-tag. Following in vitro transcription of the wild-type *CCNB3* and mutant *CCNB3* plasmids into cRNAs, they were microinjected into mouse GV oocytes. After injection, the oocytes were arrested at the GV stage in M2 medium containing milrinone for 12 h to allow sufficient translation, then washed in milrinone-free M16 medium, and cultured for 3 h to observe meiotic resumption (GVBD) or 14 h to detect PB1 extrusion. Most of the mouse GV oocytes injected with wild-type *CCNB3* cRNA could develop to MII stage. However, the oocytes microinjected with E116A, E221A and P1223L cRNA exhibited relatively lower rates of GVBD (Fig. 3A,B). Meanwhile, the PB1 rates of oocytes microinjected with all mutant cRNAs were significantly decreased compared with the negative control which did not microinjection anything (Fig. 3C,D). These results suggest that *CCNB3* is essential for meiosis during oocyte maturation and the E116A, E221A, P1223L and S1353N mutations of CCNB3 impaired the progress.

### Effects of mutations on CCNB3 expression and subcellular localization in cultured cells and oocytes

In order to examine the impacts of the mutations in vitro, an immunoblot analysis was conducted on HEK-293T cells that were transfected with either wild-type or mutant constructs. The findings indicated that the *CCNB3* mutations did not have any significant effect on the expression of CCNB3 protein level when compared to the control group (Fig. 3E,F). Under normal conditions, CCNB3 is primarily localized in the nucleus, and the predictions suggested that the mutations are situated in close proximity to the NLS or NES region (Fig. 3G). Therefore, it is hypothesized that the *CCNB3* mutations may alter its structure and subsequently modify its location. To assess the subcellular location of the pathogenic variants in vitro, we performed immunofluorescence staining in oocytes injected with wild-typr or mutant cRNA. Wild-type CCNB3 were mainly located at the nucleus. The protein variants demonstrated an increase in cytoplasmic signals, whereas S1353N mutation of CCNB3 exhibited a greater number of nucleus signals in comparison to other mutations (Fig. 3H, I). Subsequently, we conducted an immunofluorescence analysis on HEK-293T cells that were transfected with *CCNB3* wild-type or mutant plasmids. The results were consistent with those observed in oocytes, where the mutant CCNB3 was predominantly localized in the cytoplasm when compared to the wild-type CCNB3 (Supplemental Fig. 2). This led us to investigate the potential impact of changes in CCNB3 localization on its functionality.

### Lower CDK1 kinase activities, non-degradation of APC/C-CDH1 and APC/C-CDC20 inactivation caused by CCNB3 mutations

The majority of cyclins participate in oocyte maturation by binding with CDK1 to activate their kinase activities and ensure precise cellular operation. Previous research has demonstrated that *CDK1* knockout mice experience infertility due to oocyte arrest at the GV stage [19]. Downstream of CDK1, protein phosphatase 1 (PP1) and Lamin A/C, play important roles, as phosphorylation and suppression of PP1 and subsequent maintenance of Lamin A/C phosphorylation status are crucial downstream events following CDK1 kinase activity during meiosis resumption. Deletion of *CDK1* from oocytes results in an inability to phosphorylate and suppress PP1 and Lamin A/C.

**Fig. 4:**
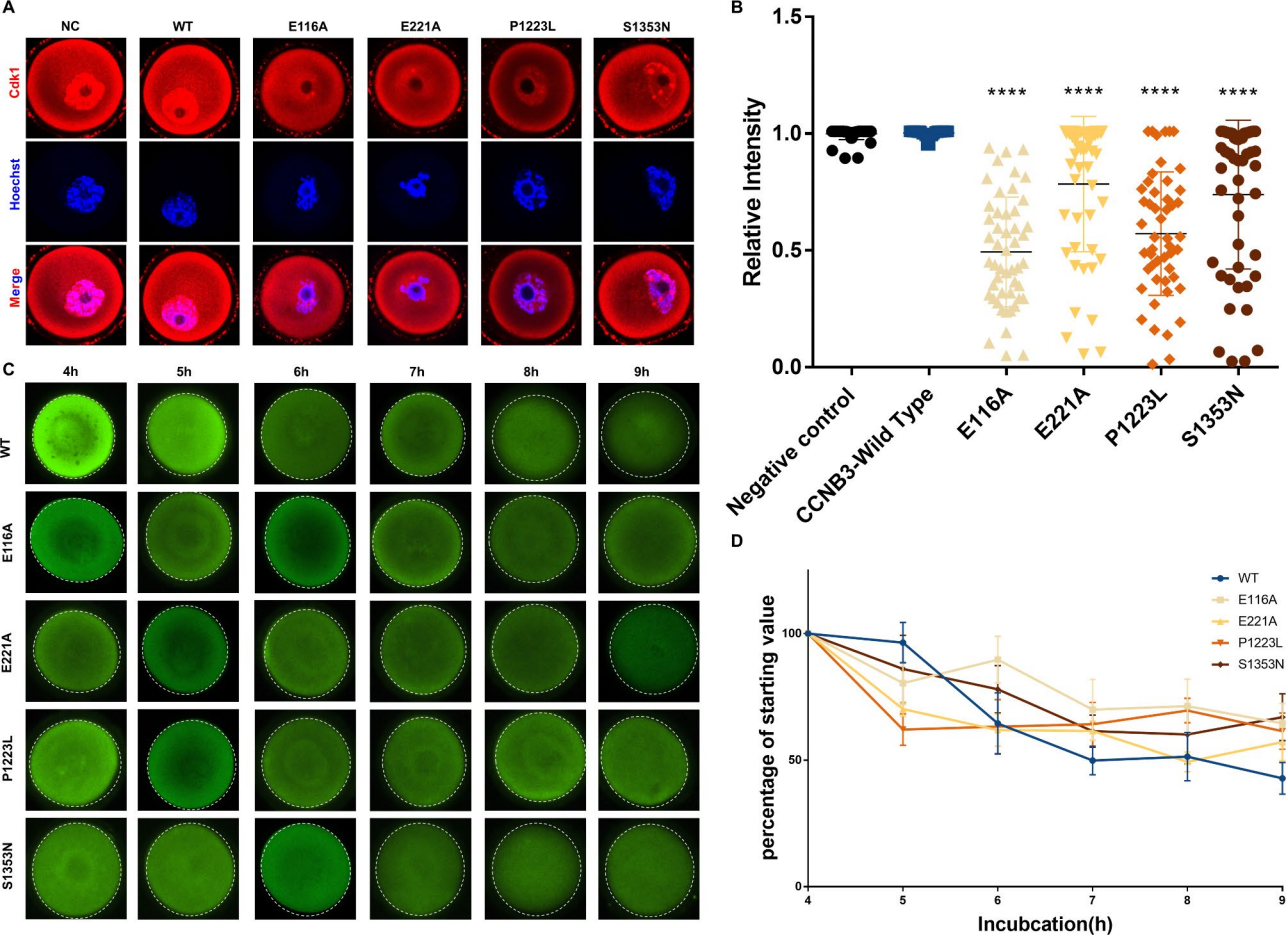
CCNB3 mutations affect the location of CDK1 and the degradation of securin. **(A)** Immunofluorescence staining of CDK1 of oocyte injected with cRNA. Each group using 40 oocytes. **(B)** Fluorescence intensities (mean ± SEM) were quantified. **(C)** Immunofluorescence measurement of securin expressed of oocyte injected with cRNA and cultured for different times. Each treatment of one time using 6 oocytes. The scale bar represents 20 μm. **(D)** Fluorescence intensities (mean ± SEM) were quantified from the indicated number of oocytes imaged

Therefore, it is hypothesized that *CCNB3* variants may contribute to the GV arrest phenotype by impacting the location of CDK1. To explore the subcellular localization of CDK1, we examined oocytes which injected *CCNB3* WT or mutant cRNAs by immunofluorescence. CDK1 was mainly localized on the nucleus and cell membrane in WT oocyte, while the protein variants exhibited increased cytoplasmic signals in the oocytes which injected mutant cRNAs (Fig. 4A, B).

**Fig. 5:**
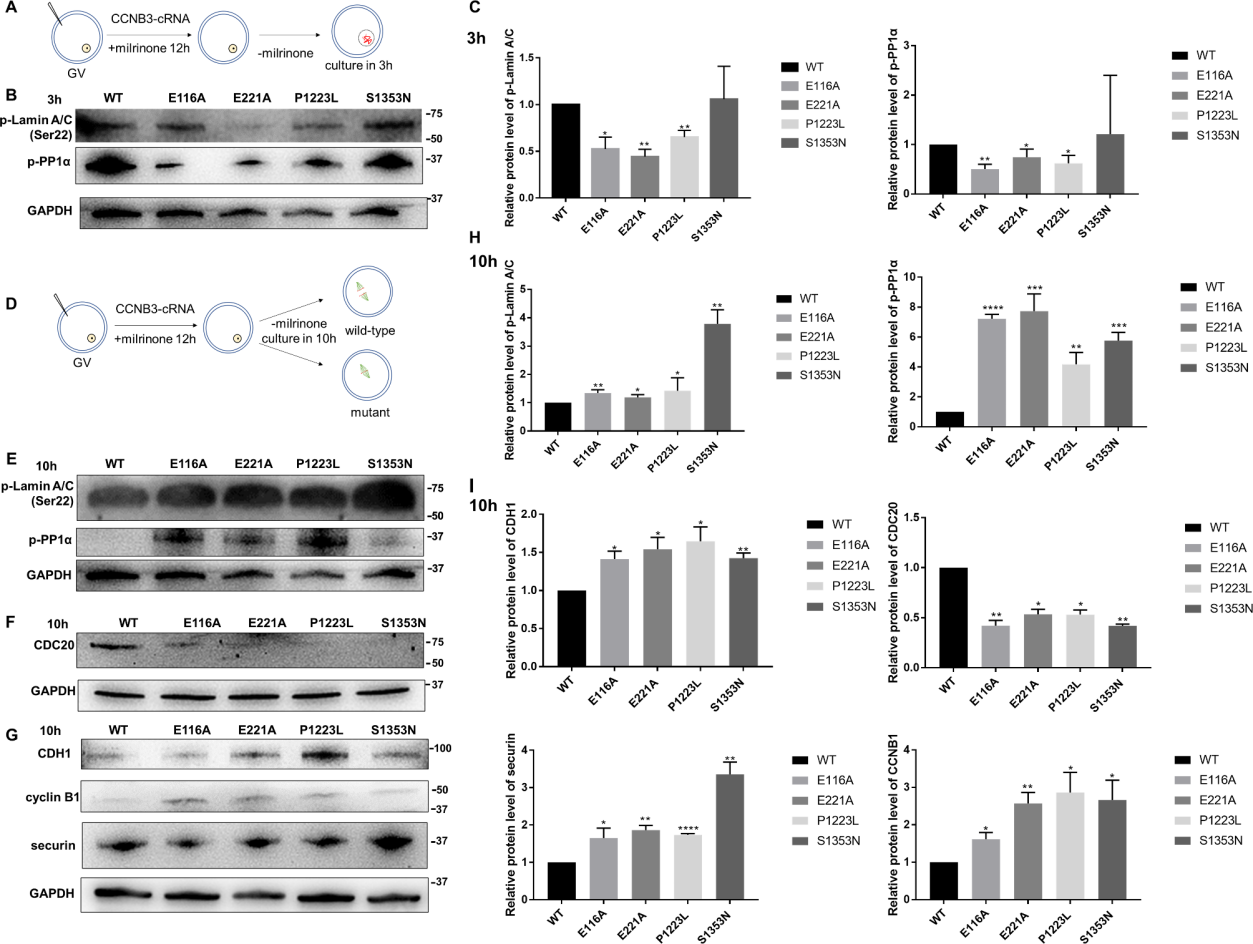
CCNB3 mutations affect the activity of CDK1 and APC/C. **(A, D)** A diagram depicting flow of mouse GV injection. GV oocytes were injected with WT or mutant CCNB3 cRNA into cytoplasm and subsequently cultured for 3 or 10 h, following collecting the oocytes. **(B-C)** Western blot analysis of oocytes injected with cRNA and cultured for 3 h in order to assay the CDK1 activity by testing the phosphorylation of Lamin A/C and PP1-α. **(E, H)** Western blot analysis of oocytes injected with cRNA and cultured for 10 h in order to assay the CDK1 activity by testing the phosphorylation of Lamin A/C and PP1-α. **(F, G)** Western blot analysis of oocytes injected with cRNA and cultured for 10 h in order to trace protein level changes of the APC/C substrates, securin and Ccnb1 protein, and the level of its activators, CDH1 and CDC20. **(I)** Relative CDH1, CDC20, CCNB1 and securin level after 10 h culture. Data are shown as the means ± SEM. For each kind of variants,n = 3 biological replicates. *P < 0.05, **P < 0.01, ***P < 0.001, **** p < 0.0001

During the resumption of meiosis, CDK1 maintains the phosphorylation status of PP1 and Lamin A/C in oocytes and CDK1 activity is increasing with the accumulation of cyclin B1. The peak of CDK1 phosphorylation of downstream substrates is at the third hour after meiotic resumption. Moreover, in order to ascertain whether the mutations we have identified exert an influence on CDK1 kinase activities and cause oocyte GV arrest, we injected *CCNB3* wild-type or mutant cRNA into GV oocytes and retrieved oocytes after a 3-hours culture period during which CDK1 activity was highly activated in normal oocytes (Fig. 5A), Our results indicate that, in comparison to wild-type controls, oocytes that overexpressed mutant *CCNB3* cRNAs, with the exception of S1353N, displayed downregulated phosphorylation of Lamin A/C and PP1-α, which suggest low CDK1 activity (Fig. 5B,C). This finding supports the notion that *CCNB3* mutations lead to oocyte GV arrest by suppressing CDK1 activity.

It has been studied in *Ccnb3*^−/−^ oocytes that they are normal until metaphase I but then fail to transit to anaphase I with defective APC/C activation, including high CDK1 activity and high cyclin and securin levels. However, in normal oocytes APC/C-CDH1 remains active through the entire prophase and prometaphase and during this stage its substrate specificity is switched to degrade CDC20. Meanwhile, CDK1-cyclin B1 decreases the activity of APC/C-CDH1 by degrading CDH1, so the degradation of CDC20 by APC/C-CDH1 gradually decreases. When CDC20 accumulates to a certain level, APC/C-CDC20 activates and degrades cyclin B1. The activity of CDK1 reaches a minimum at 10 h. Therefore, *CCNB3* wild-type or mutant cRNAs were injected into GV oocytes, which were subsequently cultured for 10 h under conditions where CDK1 activity was inhibited in normal oocytes (Fig. 5D). Analysis of the resulting oocytes revealed that overexpression of mutant CCNB3 led to upregulation of LaminA/C and PP1-α phosphorylation, indicating heightened CDK1 activity relative to wild0type control (Fig. 5E,H). Additionally, protein expression of securin and cyclin B1 was assessed via Western blotting and immunofluorescence, revealing a slower decrease in these proteins in oocytes overexpressing mutant CCNB3 compared to wild-type control. (Figures 4C and D and 5G and I).

During meiotic prometaphase I in oocytes, CDH1 directs the degradation of CDC20, which subsequently degrades with increasing CDK1 kinase activity, leading to the deactivation of APC/C-CDH1 activity and the activation of APC/C-CDC20 [20]. The mechanism by which APC/C-CDC20 inactivation results in the slow decrease of securin and cyclin B1 was investigated. Specifically, the protein levels of CDC20 and CDH1 were examined after a 10-hour culture period, revealing that variant forms of *CCNB3* led to reduced amounts of CDC20, while CDH1 levels remained high in oocytes microinjected with mutant cRNAs (Fig. 5G,I).

### Phenotypic rescue by using PP1 inhibitor and CDK1 inhibitor at corresponding periods

**Fig. 6:**
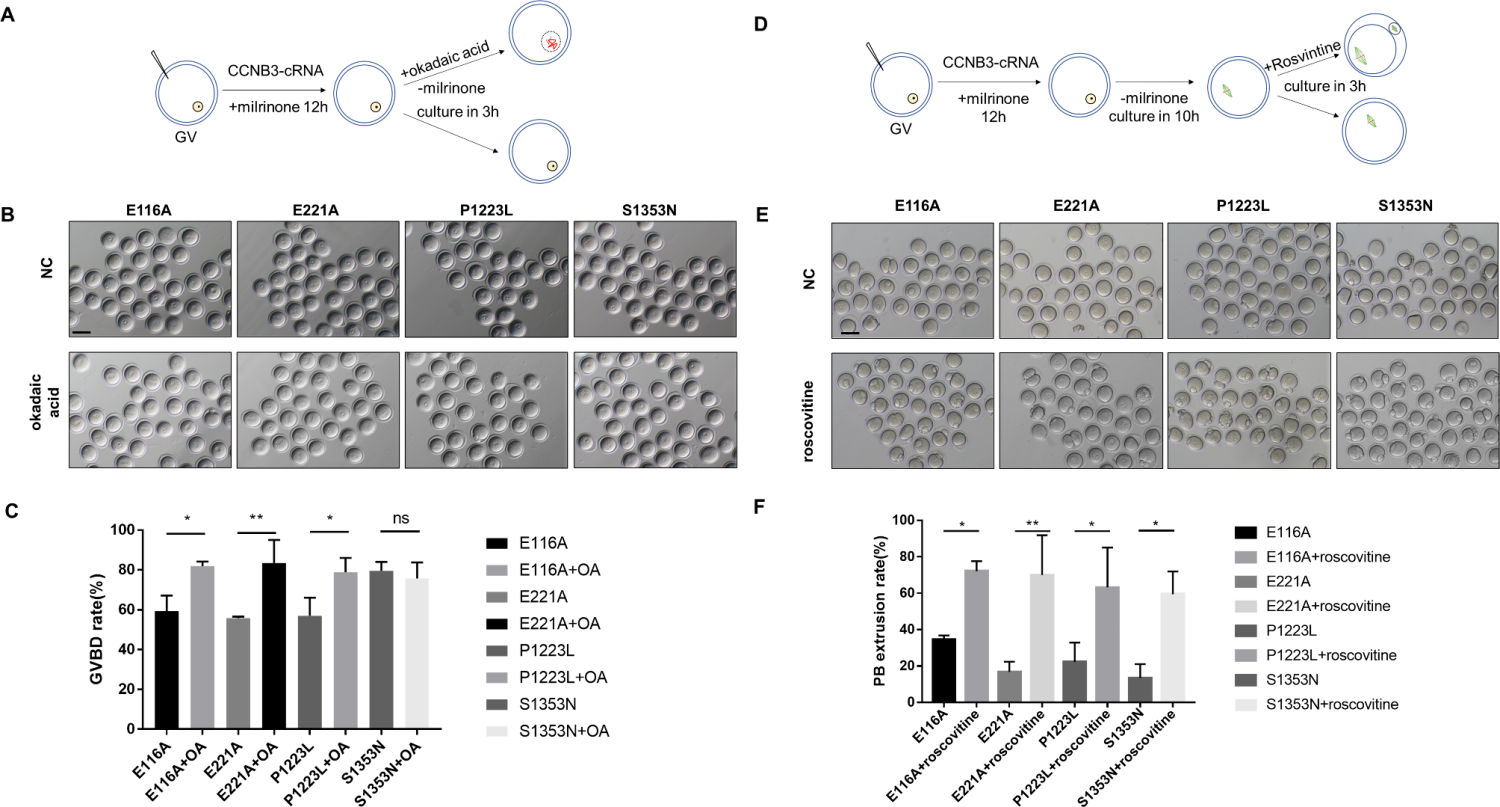
Phenotypic rescue by using PP1 inhibitor or CDK1 inhibitor at different stages. **(A-C)** diagram depicting the flow of GV oocyte culture. GV oocytes were injected with wild-type (WT) or mutant CCNB3 cRNA into cytoplasm and subsequently treated with okadaic acid(2µM) and culture for 3 h, The percentages of GVBD were scored. **(D-F)** Images of mouse GV oocytes injected with WT or mutant cRNAs and cultured for up to 10 h, and then treated with rosvintine (0.2mM) and culture for 3 h, the percentages of MII were scored. The scale bar represents 80 μm. Data are shown as the means ± SEM. For each group,n = 3 biological replicates

Following the aforementioned experimental findings, we proceeded to investigate whether the GV arrest phenotype could be remedied through chemical inhibition of PP1 phosphatase activity. To this end, we cultured oocytes that had been injected with either *CCNB3* wild-type or mutant cRNAs alongside the PP inhibitor okadaic acid (OA,2µM). As depicted in Fig. 5, treatment with OA resulted in partial resumption of GVBD in oocytes that had been injected *CCNB3* mutant cRNAs. (Fig. 6A-C). Given the high levels of securin and cyclin B1 proteins observed in oocytes injected with CCNB3 mutant cRNAs and cultured for 10 h, we posited whether inhibiting CDK activity at the onset of anaphase would suffice to induce PB extrusion in these oocytes. The CDK1 inhibitor roscovitine was utilized in this study, as it has previously been demonstrated to effectively inhibit cyclin B1-CDK1 activity in *Ccnb3*^−/−^ oocytes. Following GVBD, oocytes were treated with roscovitine (0.2mM) at the metaphase stage. Notably, the administration of roscovitine resulted in a significant increase in PB1 extrusion rates in oocytes injected with CCNB3 mutant cRNAs. (Fig. 6D-F).

## Discussion

In this study, we performed whole-exome sequencing for 68 independent infertile females that were primary infertility due to oocyte maturation arrest and their oocyte maturation rate below 40%. By filtering their WES data (shown in Supplemental Fig. 1), we identified four novel heterozygous missense mutations in the *CCNB3* gene as a possible cause of female infertility.

CCNB3 is primarily localized to the nucleus and has been implicated in the regulation of female meiosis in various organisms. In Drosophila, CCNB3 facilitates the metaphase-anaphase transition by promoting APC/C activity, independent of spindle assembly checkpoint mechanisms [21]. In mice, cyclin B3 is specifically expressed in germ cells. Knockout of *Ccnb3* resulted in infertility in female mice due to MI arrest and overexpression of the cyclin B3 gene, which interfered with the meiosis II arrest [16–18]. Oocytes lacking cyclin B3 were characteristic of insufficient APC/C activation and artificially down-regulating cyclin B1-CDK1 activity and roscovitine could rescue *Ccnb3*^−/−^ oocytes. Recently, a homozygous missense mutation (V1251D) and a homozygous protein-truncating mutation (c.4091 + 1G > A) in *CCNB3* has been reported to be association with recurrent miscarriages and aberrant meiosis [22, 23], but a causal relationship between the mutation and clinical presentation has not been definitively established, and the exact pathogenic mechanism of *CCNB3* mutations has not been determined.

The recovery of meiosis in the oocytes under normal physiological condition is linked to the activation of MPF, a complex consisting of CDK1 kinase and cyclin B1 [24–26]. The APC/C regulates the progression of the oocytes by affecting the amount of cyclin B1. Two types of APC/C activators, CDC20 and CDH1, activate APC/C at different stages of the cell cycle [27]. CDH1 directs the degradation of cyclins prior to the resumption of meiosis and is negatively regulated by CDK1 during meiotic prometaphase I [28, 29]. CDC20 plays a role in the degradation of cyclins and securin [30, 31] which function as an inhibitor of the protease separase during metaphase. Upon the destruction of securin, separase is activated and cleaves the cohesin complex ring, enabling the separation of homologous chromosomes during meiosis I.

Research has shown that APC/C-CDH1 is activated during the initial meiotic prophase and sustains oocytes in the GV stage by degrading cyclin B1 and maintaining low MPF activity. Gradually, the substrate specificity of APC/C-CDH1 shifts towards the degradation of CDC20. Ultimately, the increasing activity of CDK1 leads to this process. Subsequently, the degradation of cyclin B1 and securin facilitates the cleavage of the cohesion ring by separase, thereby enabling the segregation of chromosomes. To simulate the oocyte maturation arrest phenotype, WT or mutant *CCNB3* cRNA was microinjected into mouse GV oocytes in this investigation. The microinjection of any of the *CCNB3* mutant cRNAs resulted in maturation arrest at the MI stage, while GV arrest was induced by three of the mutant (E116A, E221A and P1223L). Given that all the pathogenic variants were situated near NLS or NES, we employed mouse oocytes and HEK-293T cells to observe the pathogenic effects of mutations. We observed that the variants induced a relocation of CCNB3 from the nucleus to the cytoplasm in vitro, accompanied by anomalous CDK1 localization and reduced kinase activity, with the exception of S1353N. Our conjecture is that this discrepancy may be attributed to the distinct effect of this mutation on localization in comparison to other mutations. Furthermore, we hypothesis that the aberrant localization of CCNB3 may impact the positioning of CDK1 in the nucleus, leading to improper functionality. This supposition was validated by immunofluorescence findings. Subsequently, CDH1 levels remained unaltered while CDC20 continued to degrade. Ultimately, the deviant level of CDC20 resulted in the deactivation of APC/C-CDC20, potentially leading to the arrest of oocyte maturation (Fig. 1). Previous studies have shown that the removal of *CDK1* in mouse oocytes impedes the phosphatase activity of PP1, resulting in GV arrest [19]. We hypothesized that intervening with Cdk1 activity during corresponding periods could potentially rescue oocyte maturation arrest caused by *CCNB3* mutations. To test this hypothesis, we utilized the PP1 inhibitor OA to culture oocytes injected with mutant *CCNB3* cRNAs and observed an improvement in meiosis resumption. Additionally, the addition of a CDK1 inhibitor during metaphase of culture oocytes proved to be effective. In future clinical ART treatment, the addition of an inhibitor may serve as a personalized therapy for individuals affected by *CCNB3* mutations.

The objective of this study is to offer genetic diagnosis and treatment for patients experiencing oocyte maturation failure. To achieve this, a clinical classification system was employed to categorize the five patients. Based on the Beall criteria [3], patient 3 was classified as type II, with all oocytes arrested at the MI stage, while the remaining patients were classified as type IV, producing oocytes that arrested at more than one stage of meiosis. It is hoped that this information will be beneficial in clinical decision-making. We suggest that if a patient with unexplained primary infertility is found in the clinic and some of her oocytes have maturation disorders, genetic testing can be performed on the patient. If the patient is found to carry a mutation in *CCNB3*, her infertility may be caused by the *CCNB3* mutation, and the mutation warrants more in-depth study. If the patient also carries other genes that have been reported to cause oocyte maturation disorders, such as *TUBB8* and *PATL2*, then further investigation is needed to determine which mutation is pathogenic or whether the two mutations act synergistically. The present study reveals that the utilization of PP1 and CDK1 inhibitors at corresponding stages can partially alleviate mouse oocyte arrest induced by CCNB3 mutation. These findings hold promise for the rescue of human arrested oocytes that have undergone aberrant APC/C activity. Nonetheless, further experimentation is required to ascertain the optimal dosage and timing of inhibitor administration prior to clinical application. Additionally, it is imperative to assess the safety of these inhibitors and their potential impact on oocyte maturation quality, including euploidy rate and developmental potential. Besides, it has been shown that injection of wild-type mRNA into oocytes which carrying pathogenic mutations can rescue oocytes [6], suggesting that we can rescue immature oocytes by injection of wild-type CCNB3 mRNA into the oocytes of patients carrying CCNB3 mutations, but the specific implementation of this protocol may require a long exploration, and extensive studies are needed for the concentration and level of mRNA injection as well as the choice of injection time points.

Notwithstanding the findings of this study, certain limitations and issues warrant further discussion. First, the study revealed phenotypic variability among affected individuals with distinct *CCNB3* missense pathogenic variants. Specifically, patient 1 and patient 2 exhibited oocytes with both GV and MI arrest, while patients 3–5 displayed MI arrest in oocytes. Furthermore, patients 4 and patient 5 experienced zygote cleavage arrest. The variability may be attributed to the location of the variants in different motifs, which play diverse roles. Secondly, the study observed that mouse oocytes expressing mutant P1223L were arrested at GV and MI stages whileas the patient carring the same mutation exhibited MI arrest only, it may be due to inter-species differences. Third, the phenotypes induced by mutant CCNB3 were found to be diverse, likely attributable to the impact of CCNB3 on CDK1, a crucial player in the entirety of oocyte maturation. As such, the mutant CCNB3 may exert its influence on the entire process of meiosis by affecting CDK1, resulting in varying oucomes. Fourthly, prior research has demonstrated that bi-allelic CCNB3 variants lead to recurrent miscarriages and aberrant meiosis, whereas the present study reveals that heterozygous variants result in GV/MI/zygote cleavage arrest. It is plausible that the in vivo environment is more favorable for oocyte maturation, but the inherent CCNB3 mutation renders these oocytes abnormal during subsequent mitosis. Our investigation also found that mouse oocytes carrying CCNB3 mutations encountered difficulties during in vitro maturation, which aligns with previous findings.

## Conclusions

In summary, we found that mutations in CCNB3 can alter the localization of its protein and highlight the effect of CCNB3 on APC/C-Cdh1, our results highlight *CCNB3* plays a critical role in human oocyte development and indicate that *CCNB3* mutations impair human oocytes maturation, especially metaphase I arrest. Meanwhile, the exploration to rescue the maturation disorder suggests an effectively therapeutic intervention for this clinical condition.

### Electronic supplementary material

Below is the link to the electronic supplementary material.

**Supplementary Material 1**

**Supplementary Material 2**

## Data Availability

ExAC Browser, http://exac.broadinstitute.org/. gnomAD, https://gnomad.broadinstitute.org/. PSORT II server, http://www.genscript.com/psort/psort2.html. SIFT, https://sift.bii.a-star.edu.sg/. PolyPhen-2, http://genetics.bwh.harvard.edu/pph2. NetNES 1.1 Server, www.cbs.dtu.dk/index.php. Moseslab, www.moseslab.csb.utoronto.ca/NLStradamus/. dbSNP, https://www.ncbi.nlm.nih.gov/projects/SNP/.
