# Supplementary figures and images for: Mutations in CCNB3 affect its location thus causing a multiplicity of phenotypes in human oocytes maturation by aberrant CDK1 activity and APC/C activity at different stages
Source: J Ovarian Res. 2023 Aug 28;16:178. doi: 10.1186/s13048-023-01229-8 (PMC10463413; doi:10.1186/s13048-023-01229-8)

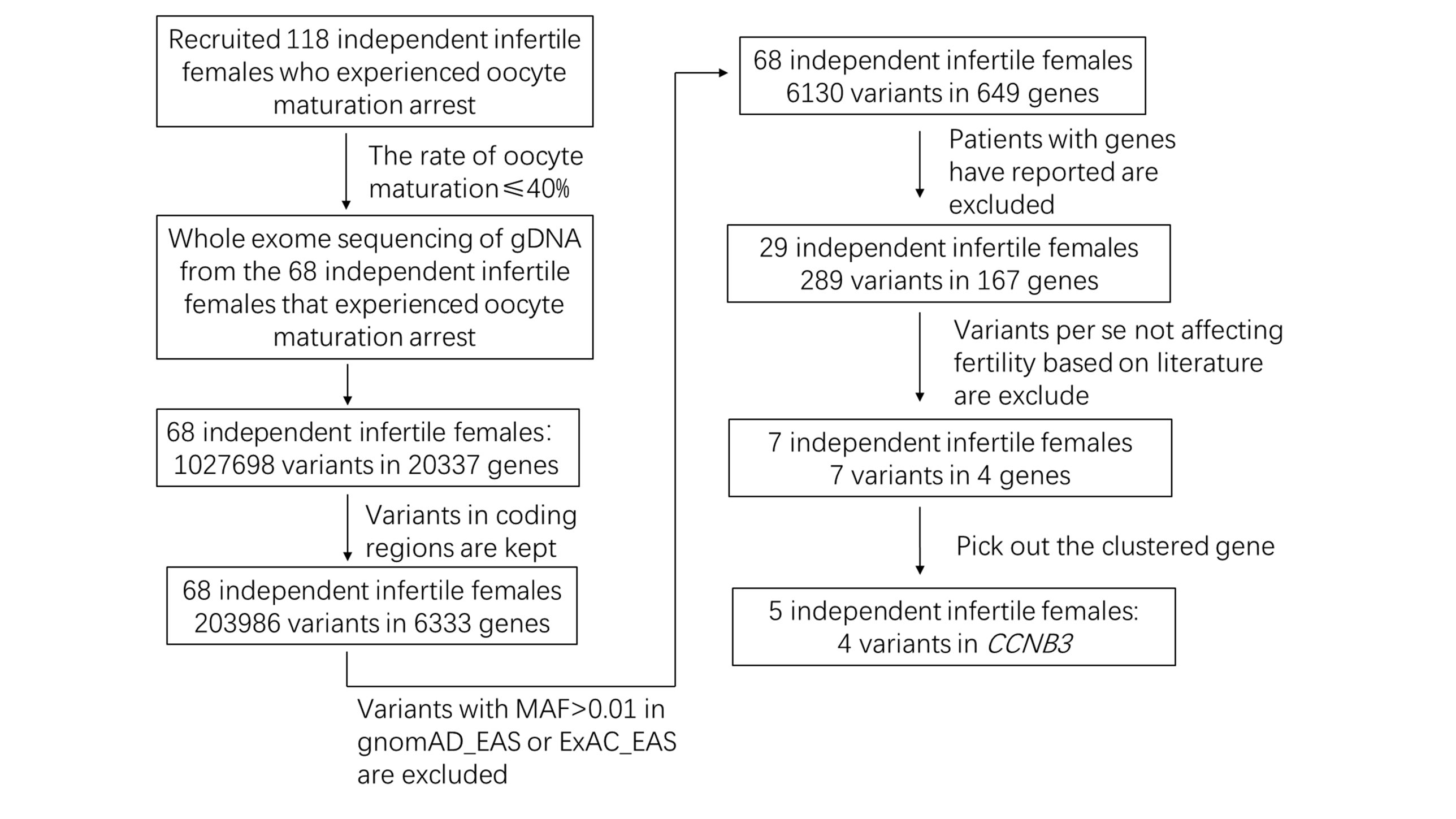

Supplement: Supplementary file 1 — Supplementary Material 1 [file 13048_2023_1229_MOESM1_ESM.png]

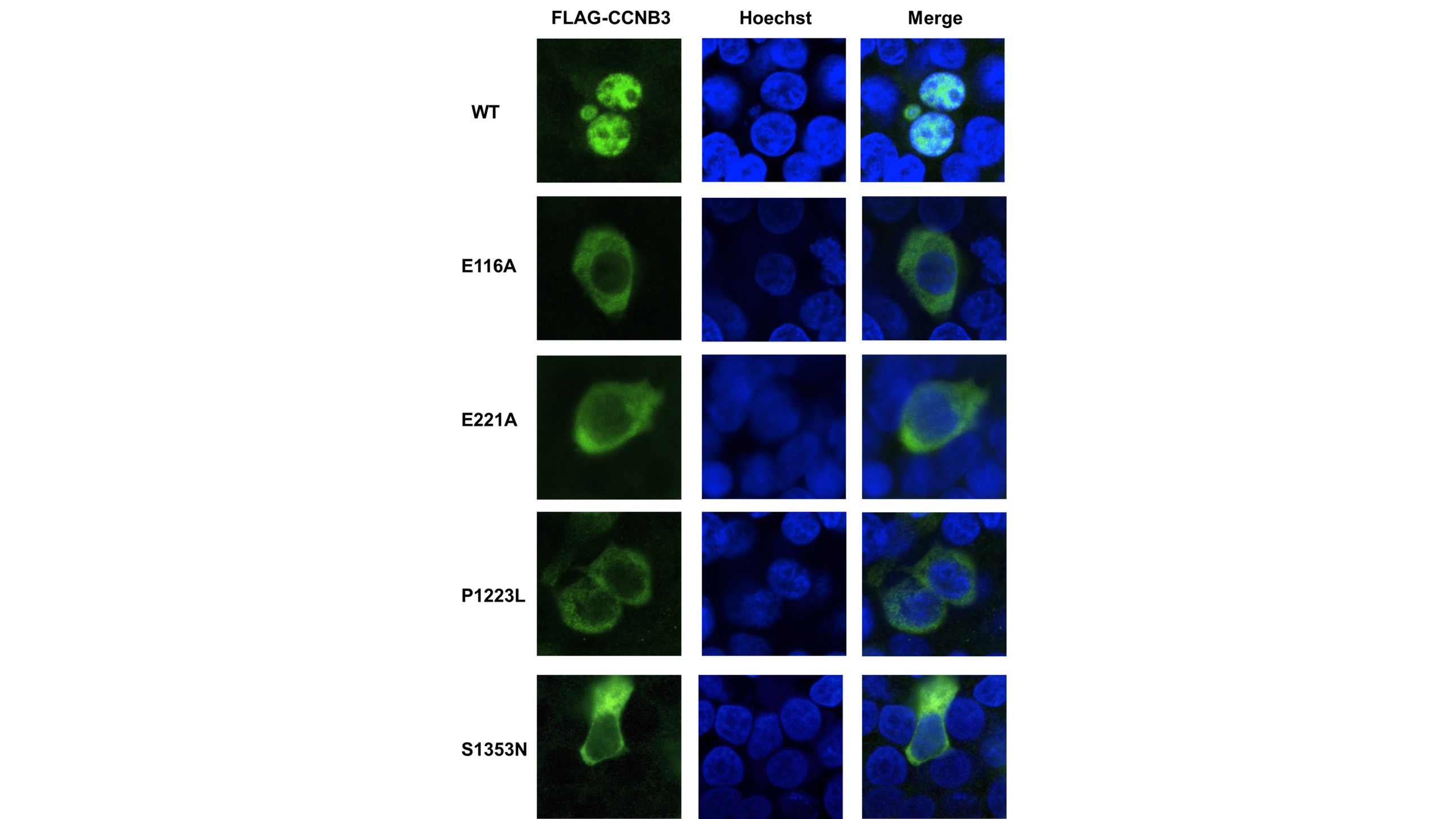

Supplement: Supplementary file 2 — Supplementary Material 2 [file 13048_2023_1229_MOESM2_ESM.png]
